# Ginsenoside RK3 suppresses osteosarcoma via PRMT5/GPX4-ferroptosis axis and enhances doxorubicin chemosensitivity

**DOI:** 10.3389/fphar.2026.1980493

**Published:** 2026-10-23

**Authors:** Zhendong Xu, Zhuang Du, Qingshan Niu, Xin Wang, Huilin Wang, Meiyan Zhang, Mozhen Liu

**Affiliations:** 1 Department of Orthopedics, The First Affiliated Hospital of Dalian Medical University, Dalian, China; 2 Department of Orthopedics, Central Hospital of Dalian University of Technology (Dalian Municipal Central Hospital), Dalian, China; 3 First Operating Room, Central Hospital of Dalian University of Technology (Dalian Municipal Central Hospital), Dalian, China; 4 Department of Orthopedics, Golmud People’s Hospital, Golmud, China; 5 Department of Neurology, Central Hospital of Dalian University of Technology (Dalian Municipal Central Hospital), Dalian, China

**Keywords:** chemoresistance, ferroptosis, ginsenoside Rk3, oteosarcoma, PRMT5

## Abstract

**Background:**

Osteosarcoma (OS), the most common primary malignant bone tumor in children and adolescents, commonly develops chemoresistance to conventional apoptosis-dependent therapies (e.g., doxorubicin) in metastatic/recurrent settings, leading to poor prognosis. Ferroptosis—an iron-dependent cell death pathway driven by lipid peroxidation—offers a promising alternative, yet clinical translation of synthetic inducers is limited by poor druggability.

**Purpose:**

This study systematically investigates the antitumor efficacy of ginsenoside RK3 in OS, explores its potential involvement in the PRMT5/GPX4-ferroptosis axis, and evaluates its potential as a doxorubicin chemosensitizer.

**Methods:**

Antitumor effects of RK3 were assessed in MG63/U2OS cells and OS xenografts. Mechanisms were explored through bioinformatics, molecular docking, co-immunoprecipitation, and ubiquitination assays. The combinatorial effects with doxorubicin (DOX) were evaluated *in vitro* and *in vivo*.

**Results:**

RK3 potently suppressed OS growth and metastasis *in vitro* and *in vivo* without significant systemic toxicity. Mechanistically, molecular docking predicted that RK3 binds to PRMT5, and RK3 treatment downregulated PRMT5 expression, which was associated with enhanced FBW7–GPX4 interaction, increased GPX4 ubiquitination and proteasomal degradation, thereby promoting iron-dependent lipid peroxidation and ferroptosis. In combination with doxorubicin, RK3 markedly enhanced chemosensitivity and tumor suppression compared to monotherapy.

**Conclusion:**

Ginsenoside RK3 inhibits OS potentially through the PRMT5/GPX4 axis to promote ferroptosis and enhances the sensitivity of tumors to doxorubicin, demonstrating a favorable safety profile. These findings suggest the PRMT5/GPX4 axis as a potential therapeutic target and position RK3 as a promising candidate for chemoresistant OS.

## Introduction

Osteosarcoma (OS), a highly aggressive malignancy predominantly affecting children and adolescents, is the most common primary bone malignancy, accounting for approximately 20% of all primary bone malignancies (Rathore and Van Tine, 2021; Siegel et al., 2025). Over the past four decades, multimodal treatment regimens—centering on neoadjuvant chemotherapy, limb-salvage surgery, and postoperative adjuvant chemotherapy—have significantly improved the 5-year survival rate of localized OS from less than 20% to 60%–75% (Bacci et al., 1990; Fernandes et al., 2024). However, patients presenting with pulmonary micrometastases at diagnosis or developing recurrence post-therapy exhibit dismal prognoses, with 5-year overall survival remaining below 30% (Beird et al., 2022). This therapeutic plateau is intrinsically linked to chemoresistance inherent to the MAP backbone regimen (methotrexate, doxorubicin, cisplatin). OS cells harbor intrinsic genomic instability and undergo therapy-driven clonal selection, evolving resistance through activation of multidrug efflux pumps (P-gp/MDR1), upregulation of antiapoptotic proteins (Bcl-2/Bcl-xL), and enhanced DNA damage repair (ERCC1/PARP1) (Nacev et al., 2022; Ning et al., 2021; Park et al., 2018; Qin et al., 2022; Tian et al., 2023; Wang et al., 2023; Zhang et al., 2015). Concurrently, cumulative toxicities—including methotrexate-induced nephrotoxicity, cisplatin-mediated ototoxicity, and anthracycline-associated cardiotoxicity—further narrow the therapeutic window (D et al., 2023; Kala and Howard, 2025; Knigh et al., 2005; Xu et al., 2025), rendering apoptosis-dependent chemotherapy ineffective in metastatic and recurrent settings. Consequently, novel non-apoptotic cell death modalities are urgently needed.

Ferroptosis, an iron-dependent regulated cell death (RCD) pathway characterized by lethal lipid peroxidation, operates independently of caspase activation and is not directly suppressed by antiapoptotic molecules such as Bcl-2, conferring a unique advantage in eliminating apoptosis-resistant tumor cells (Dixon et al., 2012; Stockwell et al., 2017). Emerging evidence implicates ferroptosis in multiple malignancies, and its targeted induction effectively circumvents chemotherapy resistance (Lu et al., 2025; Wang S. et al., 2025), such as sensitizing OS cells to cisplatin (Liu and Wang, 2019). Within this regulatory network, GPX4 serves as the critical gatekeeper by catalyzing the reduction of phospholipid hydroperoxides, GPX4 constitutes the primary defense against lipid peroxidation and ferroptotic demise (Yang et al., 2014). GPX4 is frequently overexpressed in diverse malignancies, including OS, and correlates strongly with poor prognosis (Li et al., 2023; Yang et al., 2023). Pharmacological targeting of GPX4 not only directly suppresses tumor growth but also potently sensitizes multiple cancers to conventional chemotherapeutics (Liu and Wang, 2019; Hangauer et al., 2017; Viswanathan et al., 2017). Nevertheless, most GPX4 inhibitors (e.g., RSL3, ML162) exhibit poor druggability, metabolic instability *in vivo*, and significant off-target effects (Eaton et al., 2020), limiting their clinical translation.

Previous studies on GPX4 regulation have predominantly focused on transcriptional mechanisms (e.g., the Nrf2/Keap1 axis) (Liu and Wang, 2019; Deng et al., 2024). Recently, a pivotal post-translational modification was described: protein arginine methyltransferase 5 (PRMT5) catalyzes symmetric dimethylation of GPX4 at arginine 152 (Arg152), sterically hindering recognition by the E3 ubiquitin ligase FBW7 (F-box and WD repeat domain-containing 7), thereby suppressing ubiquitin-proteasome-mediated GPX4 degradation (Fan et al., 2025). PRMT5 is commonly overexpressed in OS and associated with adverse outcomes (Li et al., 2020); however, whether it suppresses ferroptosis in OS via this mechanism remains unexplored. Although synthetic PRMT5 inhibitors (e.g., GSK3326595) have entered clinical trials, dose-limiting hematological and gastrointestinal toxicities hamper their utility (Gou et al., 2025; Newman and Cragg, 2020; Vieito et al., 2023).

Natural products have historically provided indispensable leads for anticancer drug discovery, with over 60% of approved antineoplastic agents originating from natural scaffolds or derivatives (Newman and Cragg, 2020). Ginsenoside RK3, a rare secondary metabolite derived from thermal degradation of ginsenoside Rg3 in Panax ginseng C. A. Meyer, exhibits improved bioavailability and bioactivity versus its precursor (Zhou et al., 2023). Preclinical studies demonstrate that RK3 exerts antitumor activity in hepatocellular carcinoma, non-small cell lung cancer, and glioblastoma through PI3K/AKT inhibition, apoptosis and autophagy induction, and tumor immune microenvironment remodeling (Duan et al., 2017; Qu et al., 2023; Zhang et al., 2025), with favorable safety profiles. However, whether RK3 modulates PRMT5 to induce ferroptosis in OS and enhance doxorubicin sensitivity remains unknown.

Herein, we systematically investigate the antitumor efficacy of RK3 in OS, testing the hypothesis that RK3 suppresses PRMT5 expression and activity, thereby attenuating PRMT5-mediated stabilization of GPX4, facilitating FBW7-dependent GPX4 ubiquitination and degradation, and promoting ferroptosis. We also evaluate the combinatorial efficacy and biosafety of RK3 as a chemosensitizer for doxorubicin, providing a potential candidate agent and theoretical basis for the clinical treatment of osteosarcoma.

## Results

### Ginsenoside RK3 inhibits malignant phenotypes of osteosarcoma cells *in vitro*

To evaluate the anti-tumor potential of ginsenoside RK3 against osteosarcoma, we characterised its effects on the malignant phenotypes of two human osteosarcoma cell lines, MG63 and U2OS. CCK-8 assays revealed that RK3 exerted a significant, dose-dependent suppression of cell viability (Figures 1A-C). The half-maximal inhibitory concentrations (IC50) were calculated as 125.2 μM for MG63 and 108.9 μM for U2OS cells via non-linear regression analysis (Figures 1B-D). Calcein-AM/PI double staining confirmed a progressive reduction in viable cell proportion at 0, 60, and 120 μM RK3 (Figures 1E,F). EdU incorporation assays demonstrated a dose-dependent decrease in EdU-positive cells, indicating effective arrest of proliferation (Figures 1G,H). Wound healing and Transwell invasion assays further showed that RK3 significantly delayed wound healing and markedly impaired matrix penetration in both cell lines (Figures 1I–L). Collectively, RK3 effectively suppresses OS malignant phenotypes *in vitro*, including proliferation, migration, and invasion.

**FIGURE 1:**
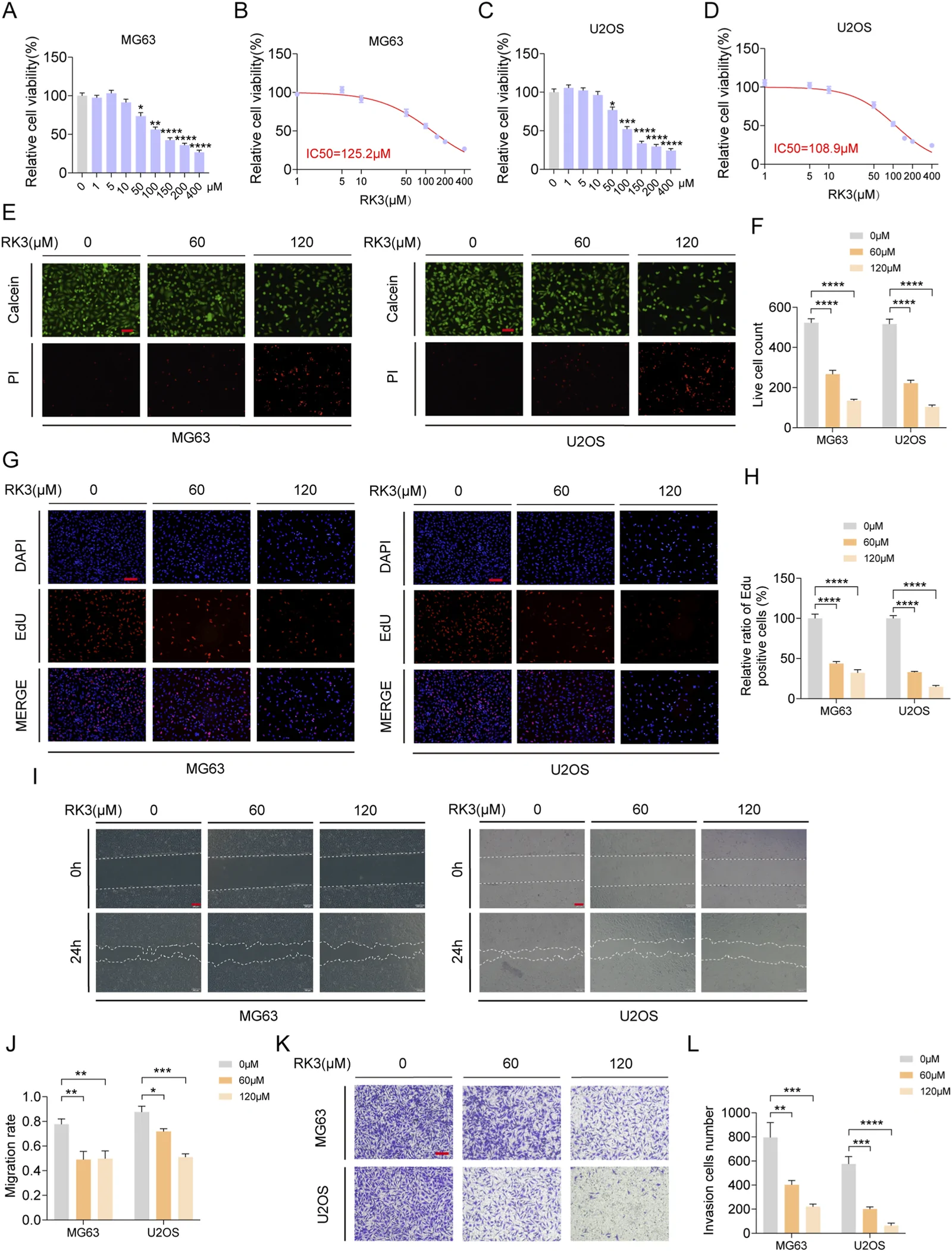
Ginsenoside RK3 suppresses the malignant phenotypes of osteosarcoma cells *in vitro*. **(A–D)** Dose-dependent inhibitory effects of RK3 on cell viability. MG63 and U2OS cells were treated with indicated concentrations of RK3 for 24 h. Cell viability was determined via CCK-8 assays **(A,C)**, and the corresponding half-maximal inhibitory concentration (IC50) values were calculated **(B,D)**. **(E,F)** Live/dead cell analysis using Calcein-AM/PI double staining. Representative fluorescence images **(E)** and quantification of live cell counts **(F)** after treatment with RK3 (0, 60, 120 μM) for 24 h. Green: live cells; Red: dead cells. Scale bar: 100 μm. **(G,H)** Evaluation of cell proliferation via EdU incorporation assays. Representative fluorescence images **(G)** and quantitative analysis of EdU-positive rates **(H)** in cells treated with RK3 (0, 60, 120 μM) for 24 h. Scale bar: 100 μm. **(I,J)** Assessment of cell migration via wound healing assays. Representative microscopic images at 0 h and 24 h **(I)** and quantification of wound closure rates **(J)**. Scale bar: 200 μm. (K, L) Evaluation of cell invasion via Transwell assays. Representative images of crystal violet-stained invaded cells **(K)** and quantification of cell numbers **(L)** after 24 h of RK3 treatment. Scale bar: 100 μm. Data are presented as mean ± SD (n = 3). *P < 0.05, **P < 0.01, ***P < 0.001, ****P < 0.0001 vs. 0 μM group.

### RK3 suppresses osteosarcoma progression *in vivo* with favourable biosafety

To validate *in vivo* efficacy and biosafety, we established a subcutaneous xenograft model. Mice received intraperitoneal RK3 (10 or 20 mg/kg) every other day for three weeks (Figure 2A). RK3 significantly retarded tumor growth, with the 20 mg/kg dose yielding the lowest tumor volumes and weights at endpoint (Figures 2B–D). To investigate whether this growth suppression was attributable to impaired cell proliferation within the tumor microenvironment, immunohistochemical (IHC) staining for Ki67 was performed. Quantitative analysis demonstrated a drastic decrease in the proportion of Ki67-positive nuclei in RK3-treated tumors compared with the extensive proliferation observed in the control group (Figures 2E,F). To assess biosafety, gross anatomical inspection of major organs (heart, liver, spleen, lung, kidney) revealed no signs of hypertrophy, atrophy, or necrosis (Figure 2F), and H&E staining demonstrated normal tissue architecture without discernible histopathological lesions or inflammatory infiltration in any examined organ (Figure 2G). To complement the histological assessment, serum levels of alanine aminotransferase (ALT), aspartate aminotransferase (AST), creatine kinase-MB (CK-MB), and cardiac troponin I (cTnI) were measured. Neither RK3 dose significantly altered these markers compared with the control group (Figures 2H–K), indicating no detectable treatment-associated elevations in the measured liver and myocardial injury markers. Together, these findings demonstrate that RK3 suppresses osteosarcoma growth *in vivo* and exhibits a favourable biosafety profile under the tested conditions.

**FIGURE 2:**
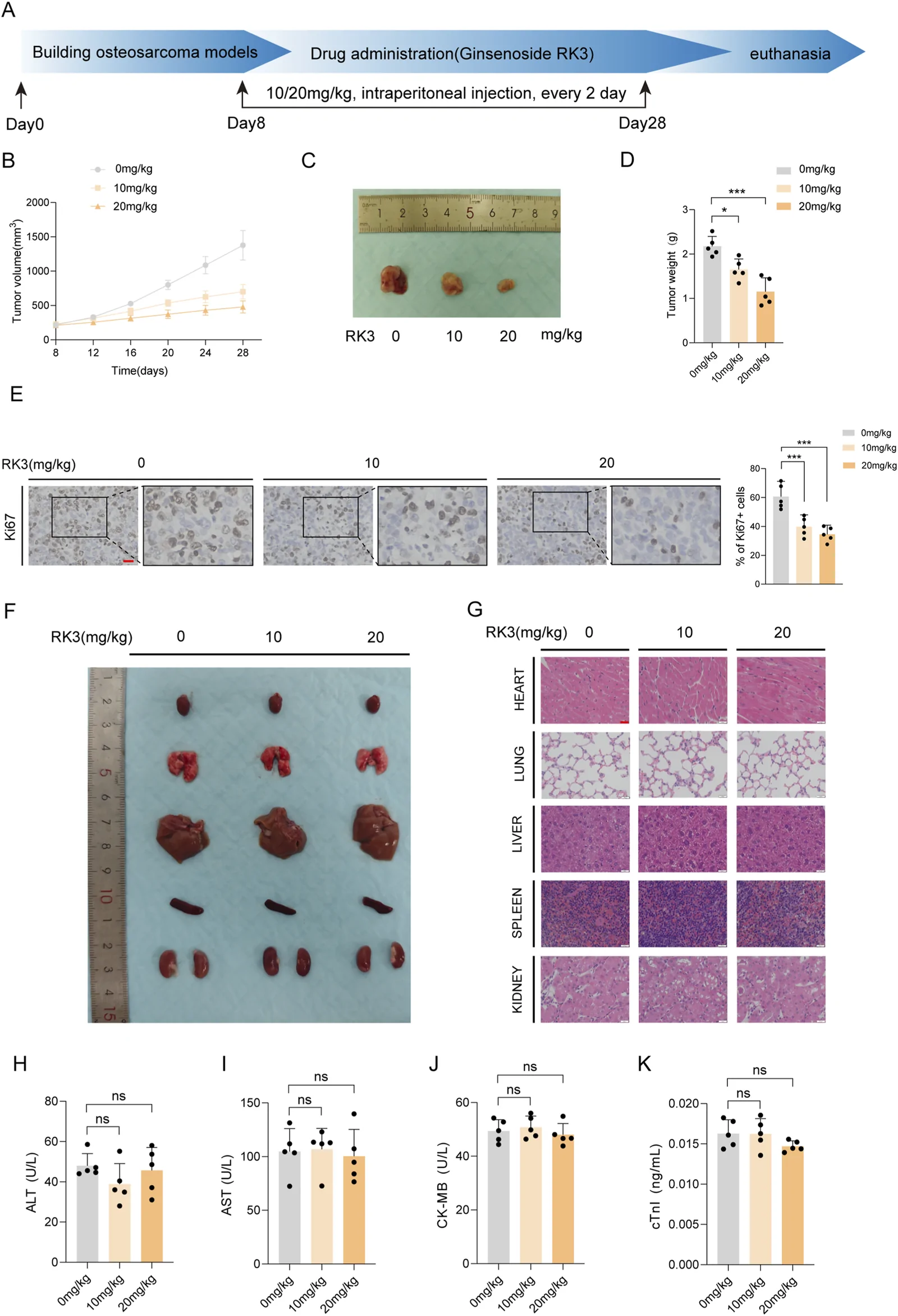
RK3 suppresses osteosarcoma growth *in vivo* and exhibits a favourable biosafety profile. **(A)** Schematic representation of the experimental design and treatment schedule. BALB/c nude mice bearing subcutaneous MG63 xenograft tumours received RK3 (10 or 20 mg/kg) or saline (control) by intraperitoneal injection every 2 days from day 8 until the experimental endpoint on day 28 (n = 5 per group). **(B)** Tumour growth curves during the treatment period. **(C)** Representative gross appearance of excised tumours at the experimental endpoint. **(D)** Tumour weights at the experimental endpoint. **(E)** Representative immunohistochemical staining of Ki67 in tumour tissues and quantification of the percentage of Ki67-positive cells. Boxed regions are shown at higher magnification in the adjacent images. Scale bar: 100 μm. **(F,G)** Representative gross appearance **(F)** and haematoxylin and eosin **(H,E)** staining **(G)** of major organs, including the heart, lung, liver, spleen, and kidney. Scale bar: 100 μm. **(H–K)** Serum levels of alanine aminotransferase (ALT; **(H)**, aspartate aminotransferase (AST; **(I)**, creatine kinase-MB (CK-MB; **(J)**, and cardiac troponin I (cTnI; **(K)**. Data are presented as mean ± SD (n = 5 mice per group). Each dot in D, E, and **(H–K)** represents an individual mouse. *P < 0.05 and ***P < 0.001 versus the 0 mg/kg control group; ns, not significant.

### Ginsenoside RK3 triggers ferroptosis in osteosarcoma cells

To dissect the mode of cell death induced by RK3, we performed pharmacological rescue experiments using Z-VAD-FMK, Nec-1, Lys05, and ferrostatin-1 (Fer-1). Fer-1 significantly and partially restored cell viability in both MG63 and U2OS cells, whereas Nec-1 and Lys05 showed no significant rescue. Z-VAD-FMK afforded modest protection in MG63 cells but not in U2OS cells (Figures 3A,B), supporting a contribution of ferroptosis while suggesting that additional mechanisms may participate. Consistent with this interpretation, RK3 increased intracellular ferrous iron (Fe^2+^), depleted glutathione (GSH), and elevated malondialdehyde (MDA) levels (Figures 3C–E). Fer-1 co-treatment significantly attenuated RK3-induced Fe^2+^ accumulation and MDA elevation and partially restored GSH levels in both cell lines (Supplementary Figures S1B–D). Lipid peroxidation was further visualized using C11-BODIPY 581/591, which showed a dose-dependent shift from reduced (red) to oxidized (green) fluorescence following RK3 treatment (Figures 3F,G). Transmission electron microscopy revealed mitochondrial ultrastructural alterations consistent with ferroptosis, including reduced mitochondrial size, increased membrane density, and loss of cristae (Figure 3H).

**FIGURE 3:**
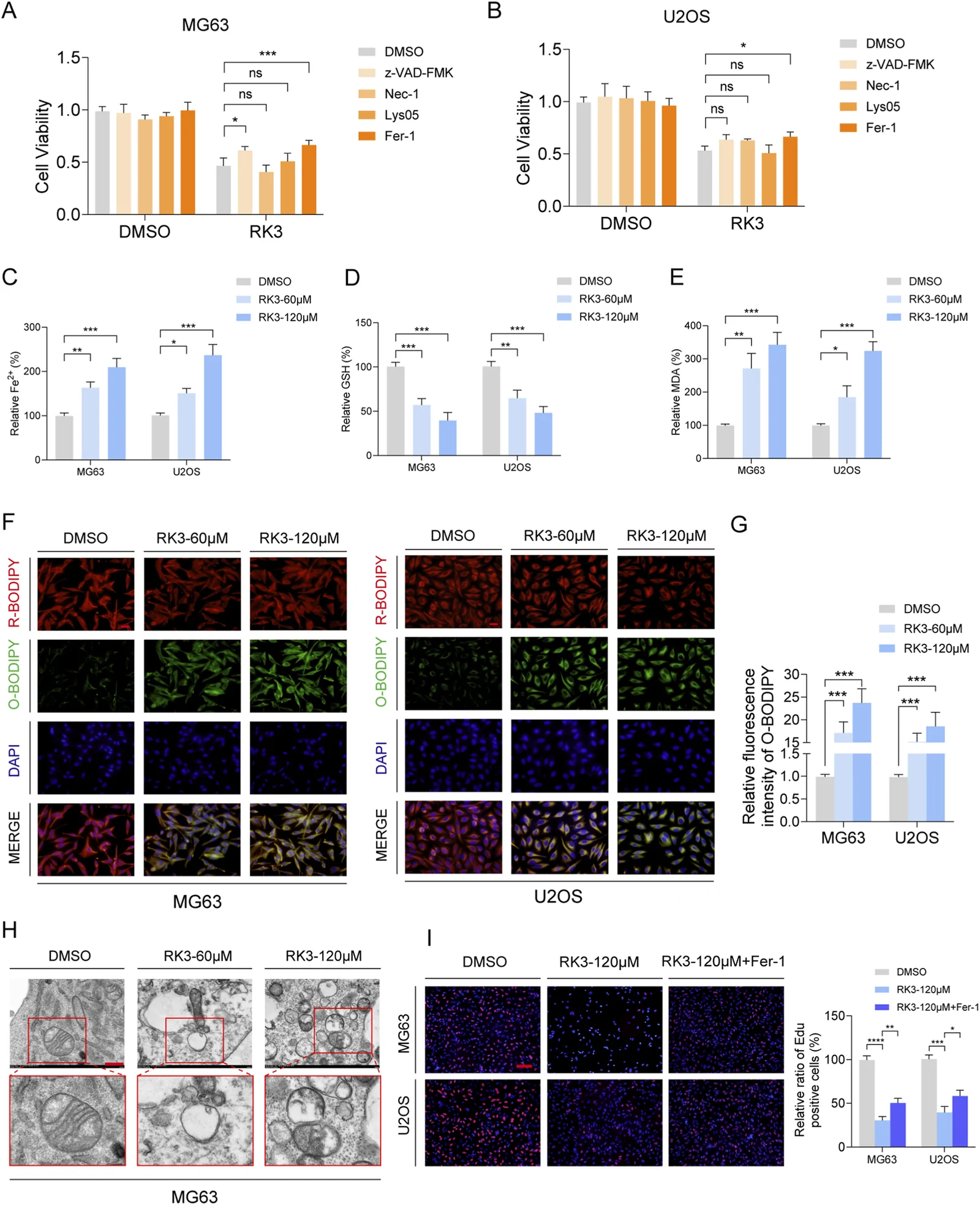
RK3 suppresses osteosarcoma malignant phenotypes by activating ferroptosis. **(A,B)** Cell death inhibitor rescue assays. **(A,B)** MG63 and U2OS cells were treated with RK3 (IC50) in the presence or absence of Z-VAD-FMK, Nec-1, Lys05, or Fer-1 for 24 h. Cell viability was assessed via CCK-8 assays. **(C–E)** Measurement of key ferroptosis biochemical markers. Intracellular levels of ferrous iron (Fe^2+^) **(C)**, glutathione (GSH) **(D)**, and malondialdehyde (MDA) **(E)** were determined in MG63 and U2OS cells treated with indicated concentrations of RK3 (0, 60, 120 μM) for 24 h **(F,G)** Lipid peroxidation was assessed using C11-BODIPY 581/591 staining. Representative fluorescence images **(F)** showing oxidized lipids (green) and non-oxidized lipids (red). Quantification of the relative fluorescence intensity ratio (Green/Red) **(G)**. Scale bar: 50 μm. **(H)** Transmission electron microscopy (TEM) observation of mitochondrial ultrastructure. The red box indicates a magnified area showing characteristic mitochondrial shrinkage, increased membrane density, and reduced or vanished cristae in the RK3-treated group. Scale bar: 500 nm. **(I)** Fer-1 reverses RK3-induced suppression of cell proliferation. EdU assays showing representative images (left) and quantitative analysis (right). Scale bar: 100 μm. Data are presented as mean ± SD. *P < 0.05, **P < 0.01, ***P < 0.001 vs. Control group.

To further characterize ferroptosis-associated molecular changes, we examined ACSL4, SLC7A11, and GPX4 protein levels. Western blotting showed that RK3 increased ACSL4 expression and decreased SLC7A11 and GPX4 levels in MG63 and U2OS cells (Supplementary Figure S1A). Consistently, immunohistochemical analysis of xenograft tumors revealed increased ACSL4 staining and reduced GPX4 staining following RK3 treatment, providing additional tissue-level evidence of ferroptosis-associated changes (Supplementary Figure S1E). Furthermore, Fer-1 partially restored EdU incorporation suppressed by RK3 in both cell lines (Figure 3I), supporting a functional contribution of ferroptosis to RK3-mediated growth inhibition. Collectively, these findings support ferroptosis as a contributing mechanism underlying the antitumor effects of RK3.

### PRMT5 is identified as a potential therapeutic target of ginsenoside RK3 in osteosarcoma

To identify molecular targets mediating RK3’s antitumor effects, we employed multi-dimensional data integration, intersecting RK3-predicted targets with differentially expressed genes from OS datasets (GSE16088, GSE225588) and the GenCLiP3 database. This approach identified six candidate genes: HSP90AA1, HDAC2, ADAM10, PRMT5, MKNK2, and SLC5A2 (Figure 4A). Subsequent qPCR and Western blotting validated that PRMT5 exhibited the most robust and consistent dose-dependent downregulation at both mRNA and protein levels following RK3 treatment in MG63 and U2OS cells (Figures 4B–D). IHC analysis of xenograft tissues confirmed a marked reduction in PRMT5 expression in RK3-treated tumors compared to controls (Figure 4H), demonstrating effective modulation of the target within the tumor microenvironment. We next investigated the potential for physical interaction between RK3 and PRMT5 using molecular docking simulations. As shown in Figure 4E, RK3 was predicted to fit into the catalytic pocket of the PRMT5 protein structure (PDB: 4GQB), forming stable hydrogen bonds, which suggests a potential direct binding affinity—although these predictions require biophysical validation. To investigate whether PRMT5 downregulation contributes to RK3-induced tumor suppression, we performed gain-of-function rescue experiments. Ectopic PRMT5 overexpression significantly attenuated RK3-induced inhibition of proliferation (EdU assay, Figure 4F) and invasion (Transwell assay, Figure 4G), with consistent results in U2OS cells (Supplementary Figures S1A,B). These data suggest that RK3 suppresses the malignant progression of osteosarcoma, at least in part, through downregulating PRMT5.

**FIGURE 4:**
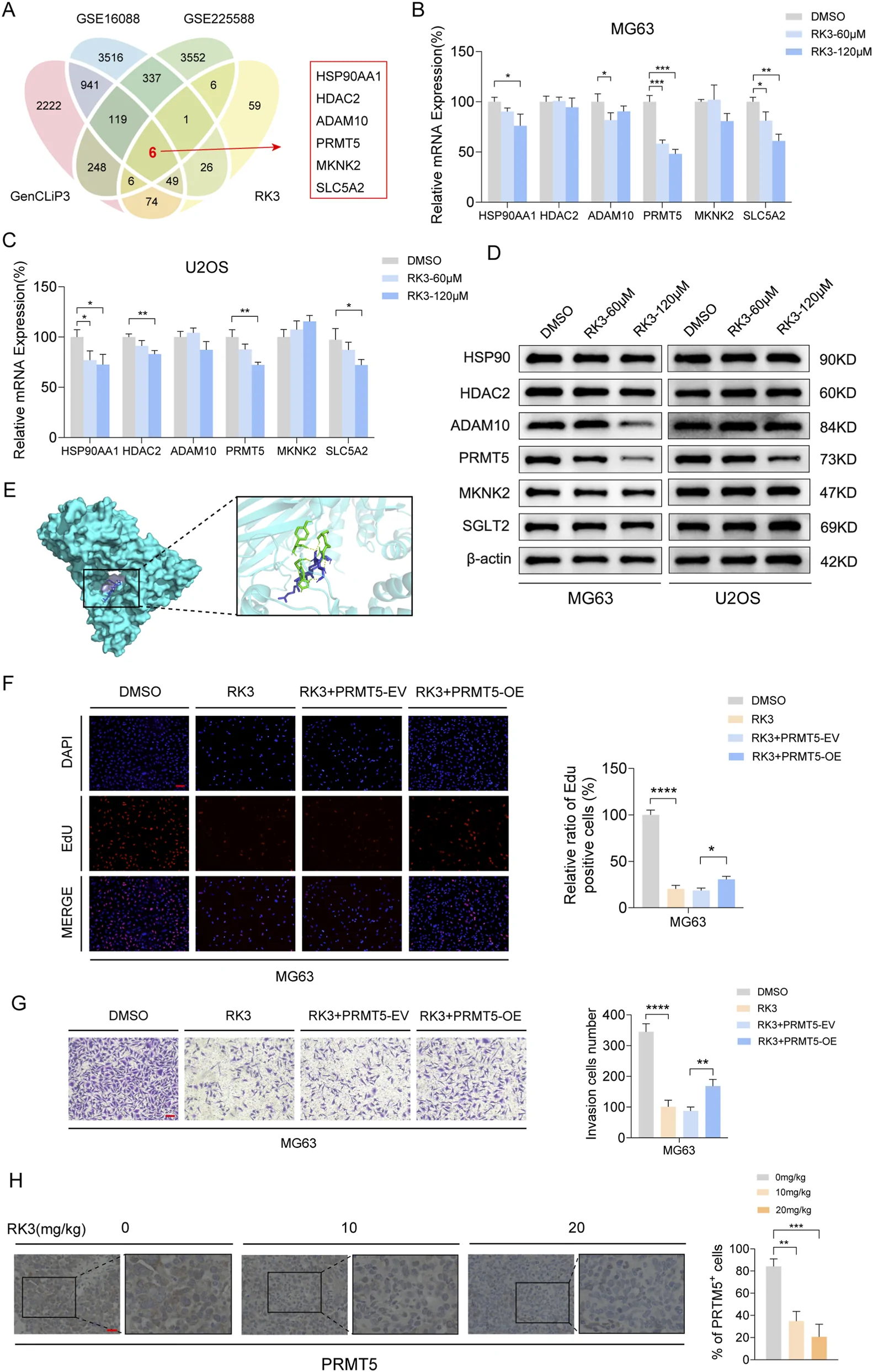
PRMT5 is identified as a functional target of RK3 in osteosarcoma. **(A)** Venn diagram showing the intersection of RK3-predicted targets, osteosarcoma-related datasets, and the GenCLiP3 database. **(B,C)** mRNA expression levels of candidate genes in MG63 and U2OS cells treated with RK3 were determined via qPCR. **(D)** Protein expression levels of candidate genes in MG63 and U2OS cells were analysed via Western blot. **(E)** Molecular docking simulation revealing the binding mode between RK3 (green stick) and the active pocket of PRMT5 protein (purple surface, PDB: 4GQB). Yellow dashed lines indicate hydrogen bond interactions. **(F,G)** PRMT5 overexpression rescue experiments. MG63 cells were transfected with PRMT5 overexpression vector (PRMT5-OE) or empty vector (PRMT5-EV) and subsequently treated with RK3 (120 μM). Cell proliferation was evaluated by EdU assays **(F)**, and cell invasion was assessed by Transwell assays **(G)**. Left: quantification; Right: representative images. Scale bar: 100 μm. **(H)** Immunohistochemical (IHC) staining of PRMT5 in tumour tissues from the xenograft mouse model. Scale bar: 100 μm. Data are presented as mean ± SD. *P < 0.05, **P < 0.01, ***P < 0.001, ****P < 0.0001.

### RK3-induced ferroptosis is associated with PRMT5 downregulation

Having identified PRMT5 as a potential target of RK3, we next examined whether PRMT5 repression is functionally linked to RK3-induced ferroptosis. To test this hypothesis, we performed rescue experiments by overexpressing PRMT5 prior to RK3 treatment. Quantitative analysis revealed that while RK3 alone triggered the characteristic ferroptotic signature (Fe^2+^ overload, GSH depletion, and MDA elevation), PRMT5-OE significantly attenuated these metabolic alterations. Specifically, PRMT5-OE reduced intracellular Fe^2+^ and MDA levels (Figures 5A,C) while partially restoring the GSH pool (Figure 5B). To visualize lipid peroxidation directly, we performed C11-BODIPY fluorescence imaging. Consistent with the biochemical findings, the strong accumulation of oxidised lipids (green fluorescence) induced by RK3 was markedly attenuated by PRMT5-OE (Figures 5D,E). These gain-of-function rescue assays establish that PRMT5 downregulation drives RK3-induced ferroptosis by disrupting redox homeostasis—promoting Fe^2+^ overload, depleting antioxidant defenses, and amplifying lipid peroxidation. This defines the RK3-PRMT5 axis as a critical modulator of ferroptotic cell death in osteosarcoma.

**FIGURE 5:**
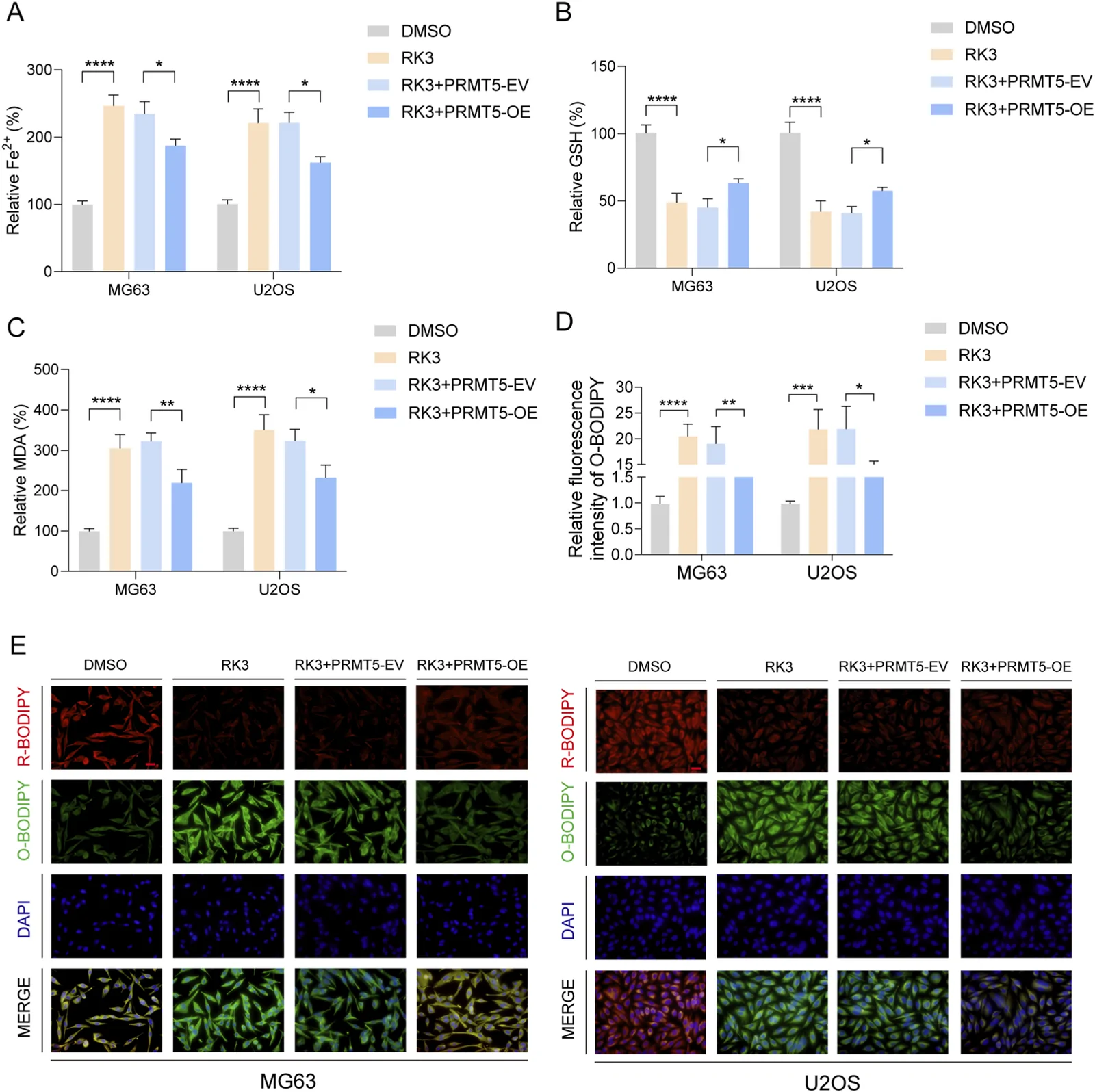
RK3 induces ferroptosis via downregulation of PRMT5. **(A–C)** MG63 and U2OS cells were transfected with PRMT5-OE or PRMT5-EV and treated with RK3 for 24 h. Intracellular levels of Fe^2+^ **(A)**, GSH **(B)**, and MDA **(C)** were measured. **(D,E)** Lipid peroxidation was assessed via C11-BODIPY staining. Quantification of the relative fluorescence intensity ratio (Green/Red) **(D)** and representative fluorescence images **(E)** showing the rescue effect of PRMT5 overexpression on RK3-induced lipid peroxidation. Scale bar: 50 μm. Data are presented as mean ± SD. *P < 0.05, **P < 0.01, ***P < 0.001, ****P < 0.0001.

### RK3 promotes GPX4 ubiquitination and degradation, which is associated with enhanced FBW7-GPX4 interaction following PRMT5 suppression

To elucidate the molecular network governing RK3-induced ferroptosis, we investigated GPX4 protein stability and ubiquitin-proteasome pathway engagement. Western blot analysis revealed that RK3 significantly reduced GPX4 protein levels, an effect rescued by PRMT5 overexpression; FBW7 abundance remained unchanged across treatments (Figure 6A), indicating that PRMT5 regulates GPX4 stability rather than FBW7 expression. Immunofluorescence staining and reciprocal Co-IP assays confirmed a physical interaction between FBW7 and GPX4 (Figures 6B–D). Treatment with the proteasome inhibitor MG132 prevented GPX4 loss regardless of FBW7 status (Figures 6E,F), establishing proteasome-dependent GPX4 turnover. CHX chase assays demonstrated that FBW7 knockdown significantly extended GPX4 half-life, whereas FBW7 overexpression accelerated its decay (Figures 6G–J), supporting FBW7 as the cognate E3 ligase for GPX4. We next explored the potential regulatory role of PRMT5 in the FBW7-GPX4 axis. Co-IP assays revealed that both RK3 treatment and PRMT5 knockdown enhanced FBW7-GPX4 binding, while PRMT5 overexpression attenuated this interaction (Figures 6K–P), indicating that PRMT5 negatively regulates FBW7-GPX4 association. At the functional level, PRMT5 overexpression mitigated RK3-induced GPX4 degradation, and this effect was reversed by MG132, implicating the ubiquitin-proteasome pathway in GPX4 turnover (Figure 6Q). CHX chase assays revealed that PRMT5 overexpression prolonged GPX4 half-life, while RK3 treatment shortened it, underscoring their opposing impacts on GPX4 stability (Figures 6R,S). Flag-GPX4 immunoprecipitation-based ubiquitination assays showed that RK3 robustly increased GPX4 poly-ubiquitination, an effect attenuated by PRMT5 overexpression (Figure 6T). Together, these data delineate a mechanism wherein RK3-mediated PRMT5 downregulation enhances FBW7-GPX4 interaction, promoting GPX4 ubiquitination and proteasomal degradation, thereby relieving GPX4-mediated suppression of lipid peroxidation and driving ferroptosis in OS cells.

**FIGURE 6:**
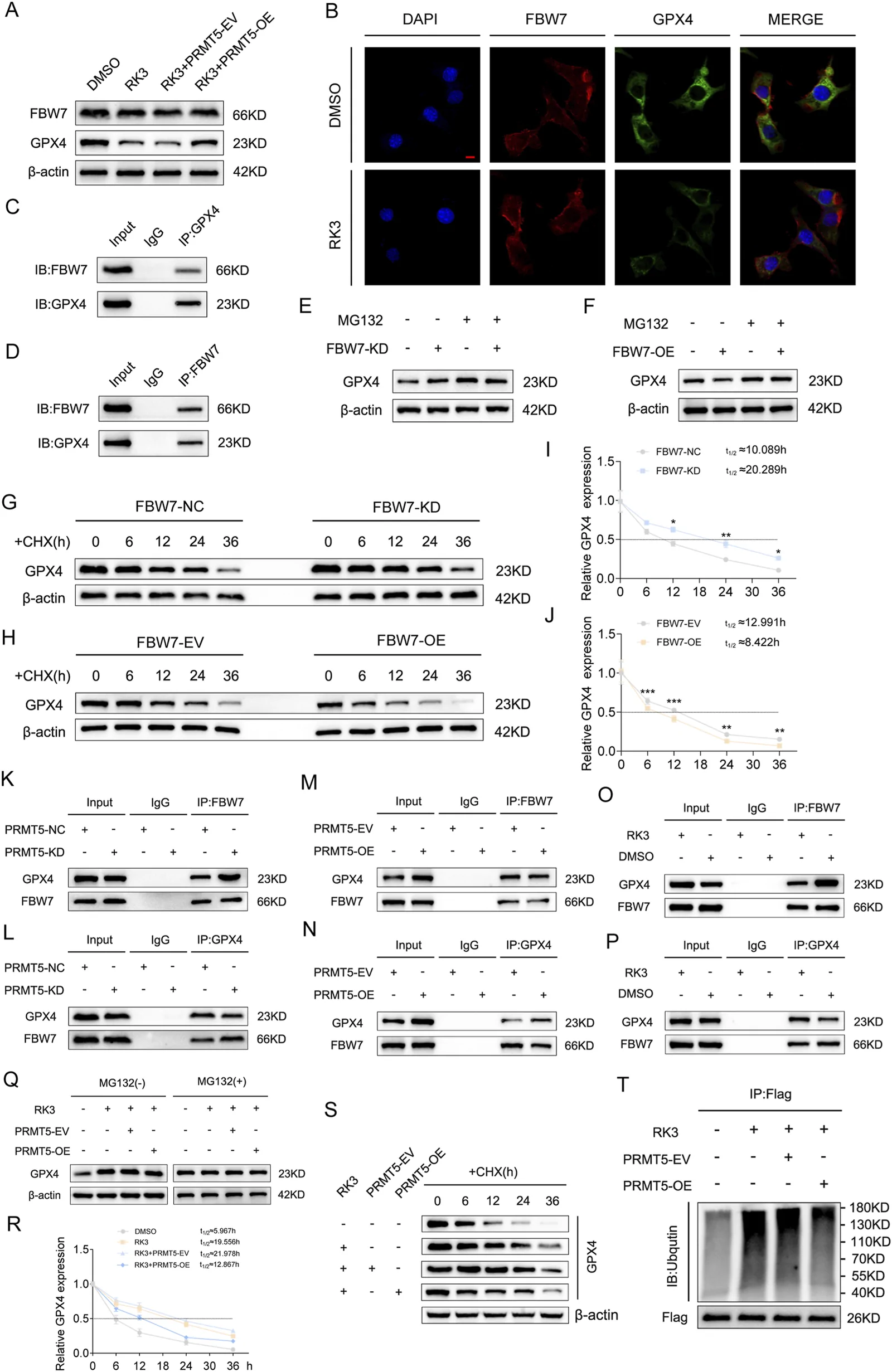
RK3 promotes GPX4 ubiquitination and degradation by enhancing the FBW7-GPX4 interaction via PRMT5 suppression. **(A)** Western blot analysis of FBW7 and GPX4 expression in MG63 cells treated with DMSO, RK3, RK3+PRMT5-EV, or RK3+PRMT5-OE. **(B)** Immunofluorescence staining showing the colocalization of FBW7 (red) and GPX4 (green) in MG63 cells. Scale bar: 10 μm. **(C,D)** Reciprocal Co-IP assays validating the interaction between FBW7 and GPX4 in MG63 cells. **(E,F)** Western blot analysis of GPX4 protein levels in FBW7-knockdown **(E)** or FBW7-overexpressing **(F)** MG63 cells treated with the proteasome inhibitor MG132. **(G–J)** Cycloheximide (CHX) chase assays. MG63 cells with FBW7-KD **(G)** or FBW7-OE **(H)** were treated with CHX (50 μg/mL) for indicated times. Quantification of GPX4 half-life is shown in **(I,J)**. **(K–N)** Co-IP assays analyzing the FBW7-GPX4 interaction in MG63 cells with PRMT5-KD **(K,L)** or PRMT5-OE **(M,N)**. **(O, P)** Co-IP assays showing the effect of RK3 treatment on the FBW7-GPX4 interaction. (Note: RK3 enhances the interaction). **(Q)** Western blot analysis of GPX4 in cells treated with RK3 and PRMT5-OE in the presence or absence of MG132. **(R,S)** CHX chase assays determining GPX4 stability in cells treated with RK3 and PRMT5-OE. Representative blots **(S)** and quantification **(R)**. **(T)** Ubiquitination assay. MG63 cells were transfected with Flag-GPX4 and Ubiquitin plasmids. GPX4 was immunoprecipitated with anti-Flag, and ubiquitination levels were detected by Western blot. Data are presented as mean ± SD. *P < 0.05, **P < 0.01, ***P < 0.001 vs. Control or indicated groups.

### RK3 enhances doxorubicin efficacy against osteosarcoma

Given that doxorubicin (DOX) is a cornerstone of OS chemotherapy but is frequently limited by resistance and toxicity, we investigated RK3 as a potential chemosensitizer. Calcein-AM/PI staining revealed that combined RK3 (120 μM) and DOX (0.5 μM) caused markedly greater cytotoxicity than either agent alone, reflected by a substantially reduced live/dead cell ratio (Figures 7A–C). To quantitatively assess the drug interaction, we performed Chou–Talalay combination index (CI) analysis at a fixed RK3:DOX molar ratio of 240:1. Six of the seven tested combinations yielded CI values below 1 in each cell line, supporting concentration-dependent synergy. At 120 μM RK3 plus 0.5 μM DOX, CI values were 0.784 and 0.555 in the analyses shown in Supplementary Figures S3A,B, respectively. (Supplementary Figures S3A,B). Combination treatment more potently inhibited DNA synthesis (EdU assay, Figures 7D,F) and cell invasiveness (Transwell assay, Figures 7E,G) than monotherapy, demonstrating collaborative suppression of OS malignant phenotypes *in vitro*. In the xenograft model, mice receiving combination therapy exhibited more pronounced tumor growth inhibition, with the lowest tumor volumes and weights among all groups at endpoint (Figures 7H–K). Further immunohistochemical analysis of Ki67 revealed that the proliferative index within the tumor tissues was maximally suppressed in the combination group (Figures 7L,M). To assess treatment-associated toxicity, we measured serum ALT, AST, CK-MB, and cTnI levels. No statistically significant differences in ALT or cTnI were detected among the groups (Supplementary Figures S3C–F). DOX significantly elevated AST and CK-MB compared with the control group, whereas RK3 co-treatment significantly attenuated the AST elevation (Supplementary Figure S3D). CK-MB was numerically lower in the combination group than in the DOX group (Supplementary Figure S3E). These findings suggest that RK3 may alleviate certain DOX-associated biochemical abnormalities, although they do not establish comprehensive systemic protection.

**FIGURE 7:**
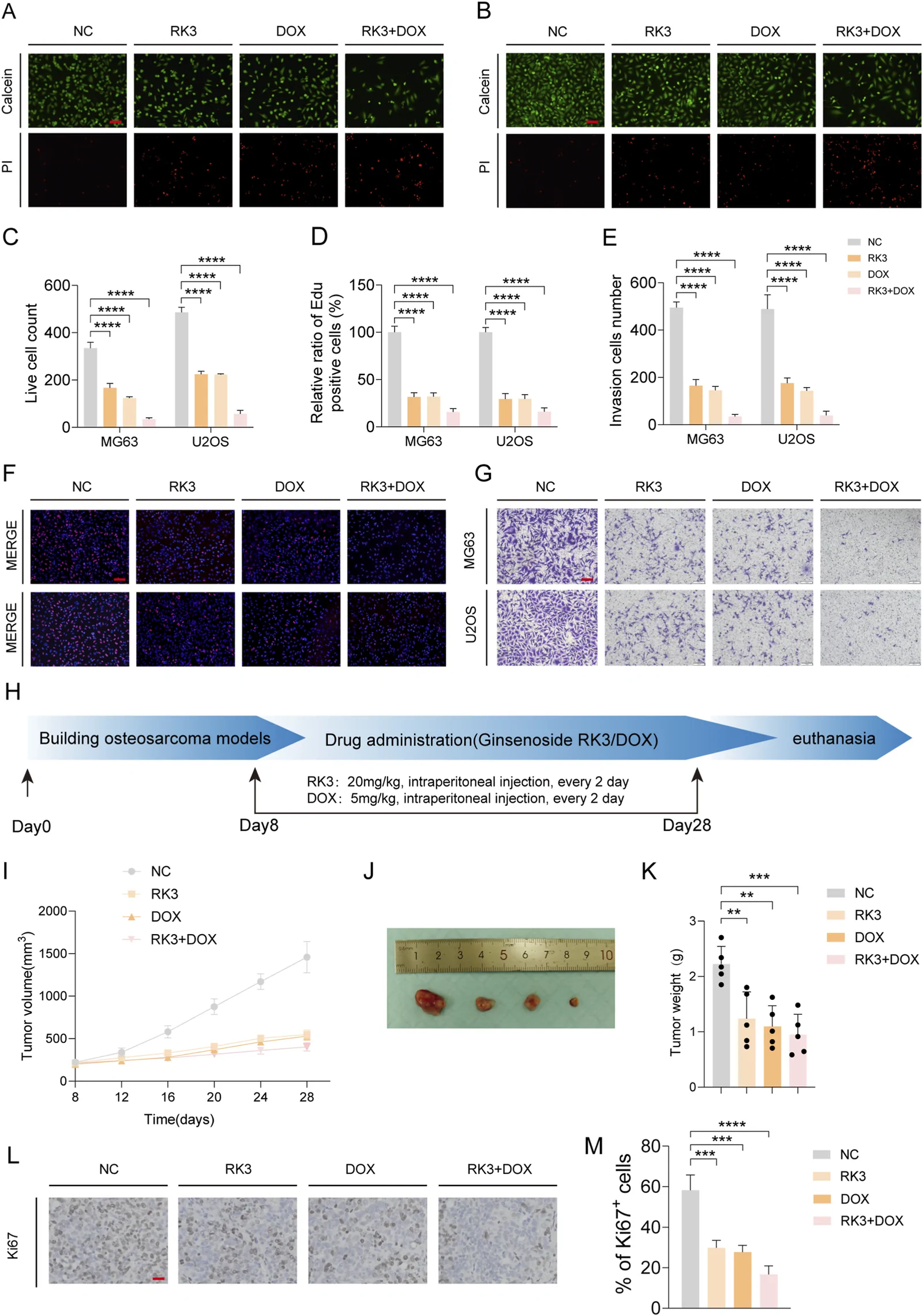
RK3 synergizes with Doxorubicin (DOX) to suppress osteosarcoma growth *in vitro* and *in vivo*. **(A–C)** Assessment of cell viability via Calcein-AM/PI double staining. MG63 and U2OS cells were treated with RK3 (120 μM), DOX (0.5 μM), or their combination for 24 h. Representative images **(A,B)** and quantification of live cell counts **(C)**. Scale bar: 100 μm. **(D,F)** Evaluation of cell proliferation via EdU assays. Representative images **(F)** and quantitative analysis **(D)**. Scale bar: 100 μm. **(E,G)** Evaluation of cell invasion via Transwell assays. Representative stained images **(G)** and quantitative analysis **(E)**. Scale bar: 100 μm. **(H)** Schematic illustration of the combination therapy regimen in the xenograft mouse model. **(I)** Tumour growth curves for each group during the treatment period (n = 5). **(J,K)** Evaluation of tumour burden at the endpoint. Representative images of excised tumours **(J)** and statistical analysis of tumour weights **(K)**. **(L, M)** Immunohistochemical staining of Ki67 in tumour tissues. Representative images **(L)** and quantitative analysis **(M)**. Scale bar: 100 μm. Data are presented as mean ± SD. *P < 0.05, **P < 0.01, ****P < 0.0001 vs. Control group.

Together, these results demonstrate that RK3 enhances the antitumor efficacy of DOX in osteosarcoma, with CI-defined synergy at selected concentrations *in vitro* and improved tumor suppression *in vivo*. The accompanying biochemical findings further support investigation of RK3 as a potential chemosensitizer with possible benefits for treatment tolerability.

## Discussion

This study provides evidence that ginsenoside RK3 suppresses osteosarcoma (OS) progression, potentially through modulating the PRMT5/GPX4 signaling axis to promote ferroptosis, while demonstrating its potential as a doxorubicin (DOX) chemosensitizer. These findings provide a foundation for exploring natural product-derived therapeutic strategies against OS.

The abysmal prognosis of metastatic/recurrent OS stems from intrinsic chemoresistance, wherein conventional apoptosis-dependent therapies fail due to OS-specific antiapoptotic mechanisms (e.g., TP53 mutations, Bcl-2 overexpression) (Ning et al., 2021; Park et al., 2018; Zhang et al., 2015). Ferroptosis, as a non-apoptotic regulated cell death modality, offers a mechanistically distinct avenue for eliminating apoptosis-resistant cells. However, synthetic ferroptosis inducers such as RSL3, ML162, and IKE face substantial clinical translation barriers due to poor metabolic stability, low bioavailability, and off-target toxicity (Eaton et al., 2020; Luo et al., 2024; Wu J. et al., 2022). Natural product-derived inducers are thus gaining prominence for their superior safety profiles. For instance, dihydroartemisinin induces ferroptosis via Fe^2+^-mediated Fenton-like reactions, and β-elemene triggers ferroptosis through SLC7A11 inhibition in KRAS-mutant colorectal cancer (Chen G. Q. et al., 2020; Chen P. et al., 2020). Our data demonstrate that RK3 exerts potent anti-OS activity associated with ferroptosis induction, without significant histopathological abnormalities at therapeutic doses in murine major organs (Figures 2, 3). Notably, only Fer-1—not inhibitors of apoptosis, necroptosis, or autophagy—reversed RK3 cytotoxicity, contrasting with prior reports of RK3 predominantly inducing apoptosis or autophagy in non-small cell lung cancer and hepatocellular carcinoma (Qu et al., 2023; Zhang et al., 2025). This cell-type specificity likely reflects OS’s mesenchymal origin, as mesenchymal-derived tumors exhibit heightened intrinsic ferroptosis sensitivity (Jiang et al., 2021).

A key contribution of this work is the identification of PRMT5 as a candidate molecular target through which RK3 promotes ferroptosis. PRMT5, a predominant type II protein arginine methyltransferase, promotes proliferation, metastasis, and stemness across multiple carcinomas by methylating histone (H3R8, H4R3) and non-histone substrates (p53, E2F1) (Barczak et al., 2020; Song and Yang, 2026). While PRMT5 overexpression correlates with poor OS prognosis and prior studies focused on its roles in cell cycle regulation and DNA repair (Li et al., 2020; Liu et al., 2024), its involvement in ferroptosis was unexplored. Through integrated multi-omics screening, molecular docking, and functional validation, we identified PRMT5 as a candidate RK3-responsive target (Figure 4E); RK3 treatment downregulated PRMT5, reducing GPX4 stability and inducing ferroptosis, consistent with Fan et al.'s discovery that PRMT5 symmetrically dimethylates GPX4 at Arg152 to block FBW7 recognition and ubiquitination (Fan et al., 2025). FBW7—a tumor suppressor and substrate-recognition component of the SCF E3 ligase complex that targets oncoproteins including c-Myc, Notch, and Cyclin E for degradation (Shimizu et al., 2018; Wang M. et al., 2025)—regulates GPX4 half-life and ubiquitination status without its own expression being modulated by PRMT5 (Figures 6G–J). This suggests that PRMT5 modifies GPX4 to impair FBW7 binding, and that PRMT5 inhibition may be less effective in FBW7-mutant or -deficient tumors (approximately 6% of human cancers (Yeh et al., 2018). Accordingly, FBW7 expression may provisionally represent a candidate predictive biomarker for RK3 and PRMT5 inhibitor efficacy—a hypothesis requiring rigorous clinical validation.

Chemoresistance critically limits long-term survival in OS. DOX, an anthracycline antibiotic and backbone of first-line OS chemotherapy (Bacci et al., 1990; Xu et al., 2025), exerts effects via DNA intercalation, topoisomerase II inhibition, and ROS generation to induce DNA damage and apoptosis (Bisht et al., 2025). However, OS cells’ genomic instability and heterogeneity drive primary/acquired resistance through multiple mechanisms (Nacev et al., 2022; Zhang et al., 2015). We demonstrate that RK3 enhances the antitumor efficacy of DOX *in vitro* and *in vivo* (Figure 7). This chemosensitization likely stems from RK3-associated GPX4 depletion, lowering the oxidative stress tolerance threshold and impairing DOX-induced ROS clearance to amplify lethal lipid peroxidation (Hangauer et al., 2017; Viswanathan et al., 2017)—a mechanism potentially distinct from conventional sensitizers. Although presented here as a proof-of-concept for preclinical efficacy, the precise quantitative nature of this interaction (e.g., synergism vs. additivity) awaits formal determination via standardized models like the Chou-Talalay CI. Crucially, DOX’s clinical utility is constrained by dose-limiting cardiotoxicity, myelosuppression, and neurotoxicity (Xu et al., 2025; Bisht et al., 2025). RK3’s favorable biosafety profile (Figures 2F,J) offers a potential strategy to reduce DOX dosing and mitigate toxicity. This generally favorable safety profile is consistent with the known characteristics of ginsenosides as a class of compounds; toxicological studies confirm their wide therapeutic windows, with some derivatives (e.g., ginsenoside Rg3) protecting against DOX-induced cardiotoxicity via oxygen radical scavenging and mitochondrial apoptosis pathway suppression.

Several limitations warrant further investigation. First, validation primarily relied on cell lines and subcutaneous xenografts; future studies should use orthotopic or patient-derived xenograft models to assess efficacy against osteolysis and metastasis within the OS bone microenvironment. Second, although molecular docking predicts RK3–PRMT5 binding (Figure 4E) and CETSA supports target engagement (Supplementary Figures S2A,B), direct physical binding remains to be established. Biophysical assays such as SPR or ITC are needed to quantify binding affinity, with kinetic measurements where applicable, while structural studies and site-directed mutagenesis could validate the proposed interaction sites. Third, the causal relationships within the proposed PRMT5–FBW7–GPX4 pathway remain incompletely established. Although RK3 reduced PRMT5 protein abundance and PRMT5 overexpression partially rescued its cellular effects, whether RK3 alters PRMT5 catalytic activity independently of protein abundance remains unresolved. Future studies measuring substrate methylation and comparing rescue by wild-type and catalytically inactive PRMT5 will be required to clarify this mechanism. Furthermore, GPX4 arginine methylation was not directly measured in this study. Therefore, although our findings support an association between PRMT5 regulation and GPX4 stability, whether RK3 influences GPX4 turnover through PRMT5-dependent methylation remains unresolved. GPX4 immunoprecipitation coupled with methylation-specific detection, together with site-specific GPX4 mutants, will be required to test this proposed mechanism. Fourth, comprehensive pharmacokinetic/pharmacodynamic and toxicology profiling is required before clinical translation. Although CI analysis supports synergy at selected concentrations *in vitro*, synergistic interactions *in vivo* and the systemic safety of the RK3–DOX combination require further evaluation. Finally, investigating combinations with methotrexate, cisplatin, immunotherapy, or PARP inhibitors may help identify effective treatment schedules and predictive biomarkers.

In summary, our data suggest that RK3 targets PRMT5 to enhance FBW7-GPX4 interaction, promoting GPX4 ubiquitination and proteasomal degradation, thereby inducing ferroptosis and suppressing OS progression *in vitro* and *in vivo*. RK3 also enhances OS sensitivity to DOX while maintaining an excellent safety profile. These findings identify the PRMT5–GPX4 axis as a potential therapeutic target and provide preclinical evidence supporting further investigation of RK3 as a standalone agent or DOX chemosensitizer for clinical translation in OS.

## Conclusion

Ginsenoside RK3 inhibits OS potentially through the PRMT5/GPX4 axis to promote ferroptosis and enhances the sensitivity of tumors to doxorubicin, demonstrating a favorable safety profile. These findings suggest the PRMT5/GPX4 axis as a potential druggable target and position RK3 as a promising candidate for chemoresistant OS.

## Materials and methods

### Cell culture and reagents

Human osteosarcoma cell lines MG63 and U2OS (Cell Bank, Chinese Academy of Sciences, Shanghai, China) were maintained in DMEM (Gibco) supplemented with 10% FBS (Gibco) and 1% penicillin/streptomycin (Gibco) at 37 °C in 5% CO_2_. All lines were STR-authenticated and confirmed mycoplasma-negative. Ginsenoside RK3 (≥98% purity), doxorubicin (DOX), ferrostatin-1 (Fer-1), Z-VAD-FMK, necrostatin-1 (Nec-1), and Lys05 were from MedChemExpress (NJ, United States); MG132 and cycloheximide (CHX) from Sigma-Aldrich (MO, United States).

### Animal experiments

All procedures were approved by the Ethics Committee of the Affiliated Central Hospital of Dalian University of Technology (Approval No. YN2025-109–01). Male BALB/c nude mice (6–8 weeks, Beijing Spf Biotechnology) received subcutaneous injection of 1 × 10^6^ MG63 cells in 100 μL PBS. Upon reaching ∼100 mm^3^, mice were randomized (n = 5/group): Control (saline); RK3 (10 or 20 mg/kg i. p., every 2 days); DOX (5 mg/kg i. p., weekly); RK3 + DOX combination. Tumor volume: V = 0.5 × length × width^2^. Animals were sacrificed after 28 days.

### Cell viability and cytotoxicity assays

Cells (5 × 10^3^/well) were seeded into 96-well plates. Following treatment, cell viability was assessed using a CCK-8 kit (Beyotime) by measuring absorbance at 450 nm. For morphological assessment of cell death, treated cells were stained with a Calcein-AM/PI Fluorescence Staining Kit (Elabscience). Live and dead cells were visualised and counted in five random fields per group using a fluorescence microscope.

### EdU proliferation assay

Cells (5 × 10^4^/well) were treated for 24 h, and proliferation was detected using an EdU Cell Proliferation Kit with Alexa Fluor 594 (Beyotime). Following fixation and permeabilization, the Click-iT reaction was performed per the manufacturer’s protocol. Nuclei were counterstained with DAPI and imaged using confocal microscopy (Olympus).

### Wound healing assay

At 90% confluence, a scratch was created in the cell monolayer. Cells were cultured in medium containing 2% FBS with indicated treatments. Wound closure at 0 and 24 h was quantified using ImageJ software.

### Transwell invasion assay

Matrigel-coated Transwell chambers (Corning, 8 μm) were used. Cells (5 × 10^4^) in serum-free medium were added to the upper chamber, with medium containing 10% FBS in the lower chamber as a chemoattractant. After 24 h, invaded cells were fixed, stained with 0.1% crystal violet, and imaged.

### Ferroptosis-related parameter assessment

Intracellular ferrous iron (Fe^2+^) levels were detected using the RhoNox-6 Staining Kit (Beyotime), with fluorescence measured at Ex/Em = 545/585 nm. Glutathione (GSH) levels were measured using a DTNB-based GSH Assay Kit (Beyotime; 412 nm). Malondialdehyde (MDA) levels were determined using a TBA reaction-based Lipid Peroxidation MDA Kit (Beyotime; 95 °C for 15 min; 532 nm). Lipid ROS was visualised using the C11-BODIPY 581/591 probe (Invitrogen), and the ratio of oxidised (green) to reduced (red) fluorescence was analysed via fluorescence microscopy.

### Transmission electron microscopy (TEM)

Cells were fixed with 2.5% glutaraldehyde at 4 °C for 6 h, post-fixed with 1% osmium tetroxide for 2 h, dehydrated in a graded ethanol series, and embedded in epoxy resin. Ultrathin sections (50–100 nm) were stained with uranyl acetate and lead citrate, and examined using a Hitachi HT7700 transmission electron microscope.

### Network pharmacology and molecular docking

RK3 targets were predicted via SwissTargetPrediction, PharmMapper, BATMAN-TCM, Super-PRED, and SEA databases. Osteosarcoma-related DEGs (|log_2_FC|>1, adj. P < 0.05) from GEO datasets (GSE16088, GSE225588) and GenCLiP3 disease genes were intersected with RK3 targets. For molecular docking, the PRMT5 structure (PDB: 4GQB) and RK3 were pre-processed using PyMOL and ChemDraw, respectively. Docking was performed using AutoDock Vina 1.1.2, and binding affinity was evaluated based on free energy and hydrogen bonds.

### Quantitative real-time PCR (qRT-PCR)

Total RNA was extracted using TRIzol (Invitrogen) and reverse-transcribed using PrimeScript RT Master Mix (TaKaRa). qRT-PCR was performed using TB Green Premix Ex Taq II (TaKaRa) on a QuantStudio 5 system (Applied Biosystems). Relative expression was calculated via the 2^(−ΔΔCt) method, normalised to β-actin (primers in Supplementary Table S1).

### Western blot analysis

Proteins were extracted using RIPA buffer (Beyotime) and quantified via the BCA method. Equal amounts of protein were separated by SDS-PAGE and transferred to PVDF membranes (Millipore). Membranes were blocked and incubated overnight at 4 °C with primary antibodies against HSP90, HDAC2, ADAM10, PRMT5, MKNK2, SLC5A2, FBW7, GPX4, Flag, Ubiquitin, and β-actin (all from ProteinTech). After incubation with HRP-conjugated secondary antibodies, bands were visualised using BeyoECL Star (Beyotime) and imaged on a ChemiDoc XRS + system (Bio-Rad).

### Gene overexpression and knockdown

Plasmids for FBW7 overexpression (FBW7-OE), knockdown (FBW7-KD), PRMT5 overexpression (PRMT5-OE), and their respective negative controls (EV/NC) were purchased from GeneChem. Cells at 70%–80% confluence were transfected with 2 μg of plasmid using Lipofectamine 3,000 (Invitrogen) according to the manufacturer’s protocol, and harvested for assays 48 h post-transfection.

### Co-immunoprecipitation (Co-IP)

Cells were lysed in IP lysis buffer containing protease and phosphatase inhibitors (Beyotime) for 30 min at 4 °C and centrifuged at 12,000 rpm for 15 min. Supernatants (500 μg protein) were incubated with specific primary antibodies (anti-FBW7, anti-GPX4, or anti-Flag, 1:100) overnight at 4 °C, followed by incubation with Protein A/G agarose beads (ProteinTech) for 2 h at 4 °C. After three washes, bound proteins were eluted by boiling in 2× SDS loading buffer and analysed via Western blot. IgG and Input served as negative and positive controls, respectively.

### Cycloheximide (CHX) chase assay

Transfected cells were treated with 100 μg/mL Cycloheximide (CHX; Sigma) for 0–36 h. Protein levels of FBW7 or GPX4 were analysed via Western blot, and band intensities were quantified using ImageJ to calculate protein half-life (GraphPad Prism).

### Ubiquitination assay

Cells were co-transfected with Flag-GPX4 and Ubiquitin plasmids, along with either PRMT5-OE or PRMT5-EV vectors (GeneChem). After 48 h, cells were treated with RK3 as indicated. Cells were lysed using IP lysis buffer containing the proteasome inhibitor MG132 to prevent protein degradation. Flag-GPX4 was immunoprecipitated using anti-Flag antibody, and ubiquitination levels were detected via Western blot using an anti-Ubiquitin antibody.

### H&E staining

Major organs (heart, liver, spleen, lung, and kidney) were harvested, fixed in 4% paraformaldehyde, and embedded in paraffin. Tissue sections (4 μm) were prepared and subjected to standard hematoxylin and eosin (H&E) staining to evaluate pathological changes and systemic toxicity. Images were acquired using an optical microscope.

### Immunohistochemistry (IHC)

Paraffin-embedded tumor sections were incubated with primary antibodies against Ki67 and PRMT5 (1:200; ProteinTech) overnight at 4 °C. Detection was performed using HRP-conjugated secondary antibodies and DAB (Beyotime), and positive cells were quantified using ImageJ.

### Statistical analysis

Data from at least three independent experiments are presented as mean ± SD. Statistical analyses were performed using GraphPad Prism 9.0 and R. Two-group and multiple-group comparisons were made using Student’s t-test and one-way ANOVA with Tukey’s test, respectively. Tumor growth curves were analysed by two-way ANOVA, and non-parametric *in vivo* data by the Mann-Whitney U test. *P* <0.05 was considered significant.

## Data Availability

The publicly available transcriptomic datasets analyzed in this study (GSE16088 and GSE225588) were obtained from the NCBI Gene Expression Omnibus (GEO) repository and are accessible at https://www.ncbi.nlm.nih.gov/geo/. All other original data generated or analyzed during this study—including raw experimental data, processed datasets, and supporting materials—are not publicly deposited but are available from the corresponding authors upon reasonable request.
