# Supplementary material for: Ginsenoside RK3 suppresses osteosarcoma via PRMT5/GPX4-ferroptosis axis and enhances doxorubicin chemosensitivity
Source: Front Pharmacol. 2026 Oct 23;17:1980493. doi: 10.3389/fphar.2026.1980493 (PMC13739687; doi:10.3389/fphar.2026.1980493)
Supplement: Supplementary file 1 [file Supplementaryfile1.docx]

**Supplementary Figure**

**Figure S1. RK3 induces ferroptosis-associated changes in osteosarcoma cells and xenograft tumours.** (A) Representative Western blots showing ACSL4, SLC7A11, and GPX4 protein levels in MG63 and U2OS cells treated with vehicle (DMSO) or RK3 (60 or 120 μM). β-actin served as the loading control. (B–D) Relative intracellular ferrous iron (Fe²⁺; B), glutathione (GSH; C), and malondialdehyde (MDA; D) levels in MG63 and U2OS cells treated with DMSO, RK3 (100 μM), or RK3 (100 μM) plus ferrostatin-1 (Fer-1). Values were normalised to the corresponding DMSO controls, which were set to 100%. (E) Representative immunohistochemical staining of ACSL4 and GPX4 in xenograft tumour tissues from mice receiving RK3 at 0, 10, or 20 mg/kg, with corresponding quantification of mean optical density (MOD). *P < 0.05, **P < 0.01, and ***P < 0.001 for the indicated comparisons.

**Figure S2. RK3 increases PRMT5 thermal stability, and PRMT5 overexpression partially attenuates the inhibitory effects of RK3 on osteosarcoma cells.** (A, B) Cellular thermal shift assay (CETSA) analysis of PRMT5 in MG63 (A) and U2OS (B) cells under vehicle (DMSO) or RK3 (60 or 120 μM) treatment conditions. Representative immunoblots and corresponding quantification show soluble PRMT5 levels following heating at the indicated temperatures (57–82 °C). (C) Representative EdU staining images and quantification of relative EdU-positive cell proportions in U2OS cells treated with DMSO, RK3, RK3 plus empty vector (PRMT5-EV), or RK3 plus a PRMT5 overexpression construct (PRMT5-OE). EdU-positive cells are shown in red, and nuclei are counterstained with DAPI (blue). Values were normalised to the DMSO control, which was set to 100%. (D) Representative Transwell invasion images and quantification of invaded U2OS cells under the same treatment conditions as in (C). *P < 0.05, **P < 0.01, ***P < 0.001, and ****P < 0.0001 for the indicated comparisons.

**Figure S3. Combination index analysis of RK3 and doxorubicin and assessment of serum biochemical markers in tumour-bearing mice.** (A, B) Combination index (CI) values calculated using the Chou–Talalay method at the indicated RK3 and doxorubicin (DOX) concentrations, with a fixed RK3:DOX molar ratio of 240:1. CI < 1, CI = 1, and CI > 1 indicate synergism, additivity, and antagonism, respectively. The dashed horizontal line denotes CI = 1. (C–F) Serum levels of alanine aminotransferase (ALT; C), aspartate aminotransferase (AST; D), creatine kinase-MB (CK-MB; E), and cardiac troponin I (cTnI; F) in tumour-bearing mice receiving vehicle control (NC), RK3, DOX, or RK3 plus DOX. Each dot represents an individual mouse (n = 5 per group). *P < 0.05 for the indicated comparisons; ns, not significant.

**Supplementary Table1.**Sequences of primers used for qRT-qPCR

| **Gene** | **Forward Primer(5'-3')** | **Reverse Primer(5'-3')** |
| --- | --- | --- |
| **HSP90AA1** | **TCAGAGCCCTTCTATTTGTCC** | **TTCTCTTTATCTTCCGCCAGT** |
| **HDAC2** | **TACGTTAATGATATTGTGCTT** | **TTATATGGCAACTCATTGGG** |
| **ADAM10** | **GATTCATCCAGACTCGTGGT** | **ACAGTCTGAATCATCCCGACA** |
| **PRMT5** | **TACTTAAGCCAGAACCGTCCT** | **TCCTCTTCTGGTACTCGGTCT** |
| **MKNK2** | **TGCAGAAGAAACCAGCCGAAC** | **CGTCTTCAAACCTGCCCGAGA** |
| **SLC5A2** | **GCCGCATCCGCCTCTACCTGT** | **CCAGCCCTCCTGTCACCGTGT** |
| **β-actin** | **CCACGAAACTACCTTCAACTCCA** | **TTTGCGGATGTCCACGTCACAC** |
